# Acceptability of HBV, HCV, and HIV screening among injured patients presenting to the adult emergency department in Blantyre, Malawi

**DOI:** 10.1186/s12879-026-13120-0

**Published:** 2026-03-19

**Authors:** Edwin Lisimba, Isabel Zgambo, Fatsani Mutala, Isaac Thom Shawa, Mulinda Nyirenda

**Affiliations:** 1 Kamuzu University of Health Sciences, Blantyre, Malawi; 2 Ministry of Health, Adult Emergency and Trauma Centre, Queen Elizabeth Central Hospital, Blantyre, Malawi; 3 Johns Hopkins Research Project, Blantyre, Malawi; 4 Biomedical and Forensic Sciences Department, University of Derby, Kedleston Road, Derby, UK; 5 Malawi Liverpool Wellcome Research Programme, Blantyre, Malawi

**Keywords:** Trauma injuries, Road traffic accidents, Bloodborne, HIV screening, Stab wounds

## Abstract

**Background:**

Trauma is among the leading causes of death and disability in Malawi and accounts for a substantial proportion of emergency admissions at tertiary hospitals. However, injured patients presenting for trauma care are infrequently screened for blood-borne viral infections, including hepatitis B virus (HBV), hepatitis C virus (HCV), and human immunodeficiency virus (HIV), resulting in missed opportunities for diagnosis and linkage to care. This study assessed the feasibility of finding new cases of HBV, HCV, and HIV among injured adult patients who presented to Queen Elizabeth Central Hospital (QECH) and evaluated the acceptability of provider-initiated opt-in testing.

**Methods:**

We conducted a cross-sectional study from October to November 2021 among injured adults (*n* = 63) seeking trauma care at the Adult Emergency and Trauma Centre (AETC) of QECH in Blantyre, Malawi. Demographic data, risk factors, and injury characteristics were collected. Blood samples were tested for HBV, HCV, and HIV using SD BIOLINE HBsAg, Rapid Anti-HCV, and Determine HIV-1/2 rapid test kits, respectively and confirmed with enzyme-linked immunosorbent assay (ELISA). Case detection rate and testing acceptability were expressed as proportions.

**Results:**

Of the 94 patients who were approached during the study period, 63 consented to participate in the study, representing a 67.02% acceptability of testing. Road traffic accident–related injuries accounted for 36.5% (*n* = 23) of the presentations, whereas non-traffic-related accidents accounted for the other 63.5%. Most refusals to participate in the study were due to fear of further blood loss and reluctance to undergo HIV testing. We found a total of 14 positive HIV cases within our study population, out of which 6 were newly diagnosed. Moreover, all cases of HBV (3 cases) and HCV (2 cases) were newly identified as well. HBV and HIV co-infection was observed in one participant.

**Conclusion:**

A considerable proportion of injured patients presenting to the AETC had undiagnosed blood-borne viral infections, with reasonably high acceptability of testing. Incorporating routine HBV, HCV, and HIV provider-initiated testing into trauma care settings could improve early detection, linkages to care, and infection prevention strategies.

**Clinical trial number:**

Not applicable.

**Supplementary Information:**

The online version contains supplementary material available at 10.1186/s12879-026-13120-0.

## Introduction

Bloodborne viral (BBV) infections such as hepatitis B virus (HBV), hepatitis C virus (HCV) and human immunodeficiency virus (HIV) remain major global public health challenges, particularly in low- and middle-income countries where healthcare resources are limited, and exposure risks are high. A substantial proportion of individuals living with HIV, HBV, and HCV remain unaware of their serostatus, which contributes to ongoing transmission and adverse clinical outcomes [1–3]. Early diagnosis plays a critical role in improving disease prognosis through the timely initiation of antiviral therapy. HCV infection is often curable, while HIV and HBV can be effectively controlled with long-term antiviral treatment [4]. These viral pathogens share similar routes of transmission, including exposure to infected blood and body fluids, unsafe medical practices, sexual contact, and perinatal transmission, resulting in frequent cocirculation within the same populations. Trauma patients represent a particularly vulnerable group due to the increased likelihood of contact with blood products, invasive procedures and emergency surgical interventions, which may heighten the risk of both acquisition and transmission of blood-borne infections within healthcare settings. Emergency and trauma settings, therefore, represent strategic points for identifying undiagnosed infections and facilitating linkages to care.

Chronic HBV infection remains a major cause of liver cirrhosis and hepatocellular carcinoma (HCC), particularly when exposure occurs in infancy or early childhood [5]. In Malawi, HBV contributes significantly to liver-related morbidity and mortality [6], highlighting the importance of screening in high-risk populations, including patients presenting with trauma. HCV infection frequently progresses silently, ultimately causing liver fibrosis, cirrhosis, or HCC [5]. Transmission is primarily blood-borne, placing both patients and healthcare workers at risk in trauma care settings. Host factors, including lipid metabolism and immune responses, may confer resistance in some exposed individuals [7]. In Southern Africa, HCV prevalence is low, predominantly affecting older adults and accounting for fewer than 5% of liver disease cases, indicating historical rather than ongoing transmission [8].

A 2018 systematic review conducted in Malawi reported a pooled seroprevalence of 8.1% for HBV and 7.3% for HCV, highlighting a substantial burden of viral hepatitis in the country [6], whereas a seroprevalence of 6.0% for HBV was reported by Nkhata et al. [9], and none by Chipetah et al. [10]. In the same year, approximately one million people were living with HIV in Malawi. Coinfection with HIV worsens the clinical course of HBV and HCV by accelerating liver disease progression, increasing susceptibility to opportunistic infections, and complicating clinical management [11, 12]. Given the high local prevalence of these infections, healthcare workers managing trauma patients at Queen Elizabeth Central Hospital (QECH) are at increased risk of occupational exposure. Transmission of blood-borne pathogens may occur through percutaneous injuries, mucocutaneous contact with blood or body fluids, and exposure to other potentially infectious fluids [13–16].

Malawi is currently experiencing a significant rise in trauma-related injuries, with road traffic accidents responsible for over 48% of cases at tertiary hospitals like QECH, while Interpersonal violence and falls also contribute significantly to emergency admissions [17]. Despite this rising trend, data on the burden of undiagnosed HBV, HCV and HIV infections among trauma patients is scarce. Understanding the extent of these infections in trauma settings is essential for informing infection prevention strategies, guiding screening policies, protecting healthcare workers, and improving patient management. This study aimed to evaluate the case detection yield of HBV, HCV, and HIV infections among trauma patients presenting to QECH in Malawi and to assess the feasibility of implementing routine testing.

## Methods

### Study setting

This study was conducted at the Adult Emergency and Trauma Centre (AETC) of Queen Elizabeth Central Hospital (QECH) in Blantyre, Malawi. Rapid serological assays were performed at the Kamuzu University of Health Sciences (KUHeS) laboratory with confirmatory testing at the Johns Hopkins Research Laboratory. The AETC functions as a tertiary referral centre for trauma patients from across the southern region of Malawi and operates a dedicated trauma care pathway. On average, 10–20 trauma patients present to the AETC within a 24-hour period, with higher patient volumes observed on weekends. The trauma room is appropriately structured to ensure patient privacy during history taking, clinical examination, specimen collection, treatment, and counselling.

### Study population

The study included trauma patients aged between 17 and 65 years who presented to the AETC during the study period and who were able to provide informed consent; patients who were unable to consent were excluded. Data were collected between October and November 2021. The minimum required sample size was estimated using a standard formula for prevalence studies with a 95% confidence level, an assumed prevalence of 9%, and a 5% margin of error, yielding 126 participants. Due to logistical constraints, 63 participants were ultimately enrolled.

### Data collection procedures

Eligible participants were approached and provided written informed consent before enrolment. Data were collected using a structured questionnaire specifically designed for this study (Supplementary File 1) and administered by the investigators. The questionnaire captured demographic characteristics, medical history, details of injury, and clinical diagnosis. Venous blood samples (5 mL) were collected into red-top tubes (EREZ Labmed, LOT 201208, South Africa) and transported in a cooler box to the KUHeS laboratory. Samples were allowed to clot and subsequently centrifuged at 3,000 rpm for 10 min. Serum was aspirated using Pasteur pipettes into cryovial tubes (SARSTEDT AG & Co. KG, LOT 1083321, Germany) and stored at − 20 °C until analysis.

### Laboratory testing

Rapid serological testing for HIV, hepatitis B virus (HBV), and hepatitis C virus (HCV) was performed using validated commercial test kits in accordance with the manufacturers’ instructions. HIV testing was conducted using the Determine HIV‑1/2 SET (Abbott, LOT 11666k200R, USA), with 50 µL of serum applied and results interpreted between 15 and 60 min. This assay has documented a sensitivity of 100% and a specificity of 99.7%. Hepatitis B surface antigen (HBsAg) testing was performed using the SD BIOLINE HBsAg WB test (Standard Diagnostics, Inc., LOT 01EDF003A, Republic of Korea), which uses 100 µL of serum and is read at 20 min. Published evaluations report a sensitivity and specificity of ≥ 99%. Anti‑HCV antibodies were detected using the Advanced Quality Rapid Anti‑HCV Test Card (InTec Products, Inc., LOT GJ20070557, China), in which 10 µL of serum and 100 µL of diluent are added, and results are read within 15–20 min, with specificity and sensitivity exceeding 99%. As such, all rapid tests had high positive predictive values and negative predictive values suitable for testing in our study population. For each rapid test, the presence of red bands in both the control and test windows indicated a reactive result, while a single red band in the control window indicated a non‑reactive result.

All samples were subsequently transported to the Johns Hopkins Research Laboratory for confirmatory testing using enzyme-linked immunosorbent assays (ELISA), performed according to standardised laboratory operating procedures. Confirmatory assays included the GS HIV Combo Ag/Ab EIA (BIO-RAD, LOT 126RJJ-02, USA), a fourth-generation test detecting HIV-1 p24 antigen and antibodies to HIV-1/2, with reported sensitivity > 99.9% and specificity > 99.5%; the GS HBsAg EIA 3.0 (BIO-RAD, LOT 139RCC-05, USA), with sensitivity and specificity ≥ 99%; and the ORTHO HCV 3.0 ELISA with Enhanced SAVe (LOT EXE 320, USA), a third-generation assay exhibiting > 99% sensitivity and specificity. All ELISA runs included manufacturer-provided positive and negative controls to ensure assay integrity and reproducibility.

### Data analysis

The case detection rates for HIV, HBV, and HCV infections among participants with previously unknown serostatus were calculated and expressed as percentages. Acceptability of testing was assessed as the proportion of approached patients who consented to participate. Categorical variables were compared using Fisher’s exact test. Multivariable logistic regression analyses were performed to identify patient characteristics independently associated with each viral infection. Statistical significance was defined as a p-value < 0.05, and results were reported with corresponding 95% confidence intervals. Data analysis was conducted using R version 4.3.2.

## Results

### Participant recruitment, enrolment, and acceptability of testing

**Fig. 1:**
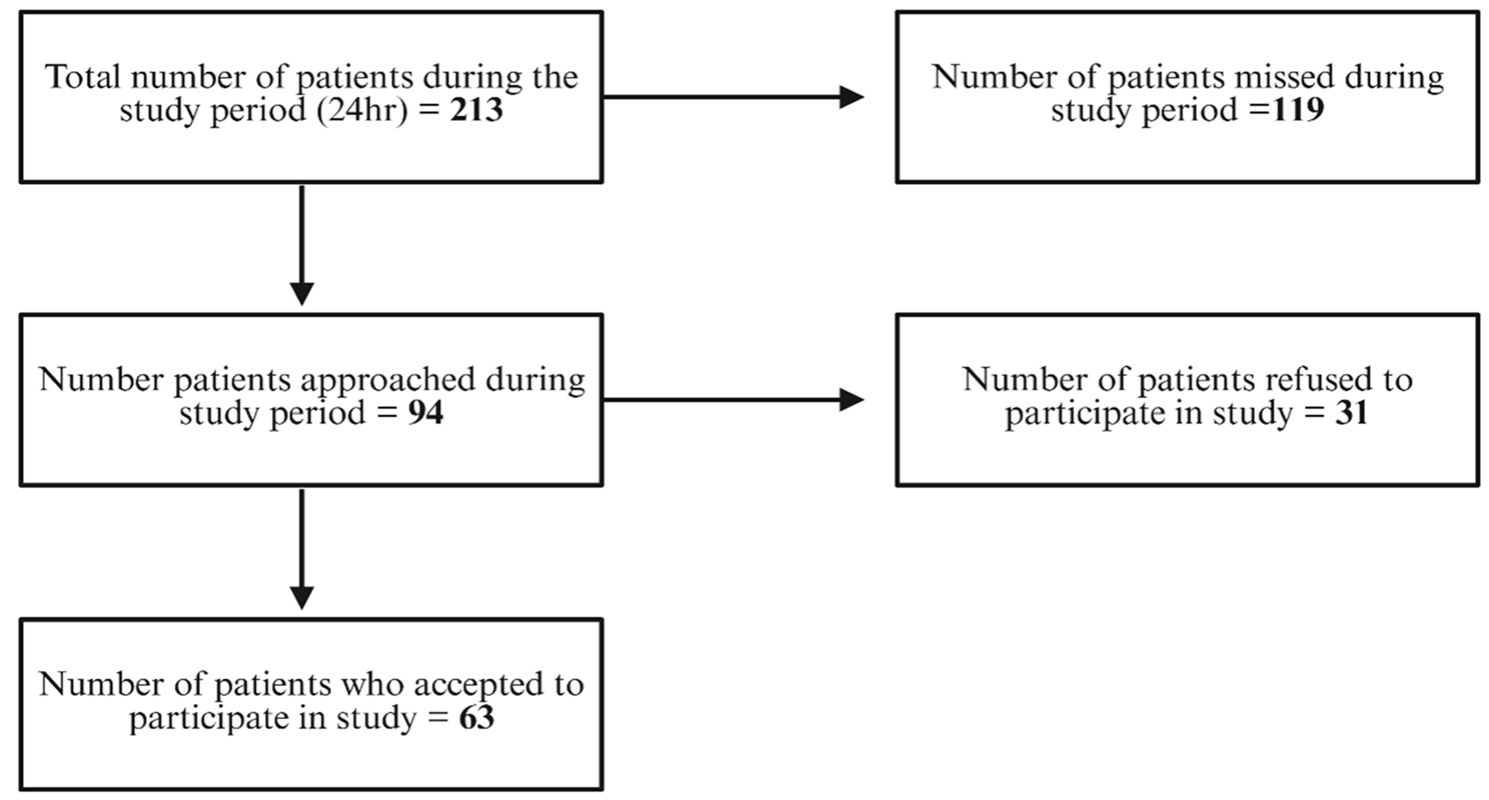
Flowchart depicting the recruitment process of study participants

**Fig. 2:**
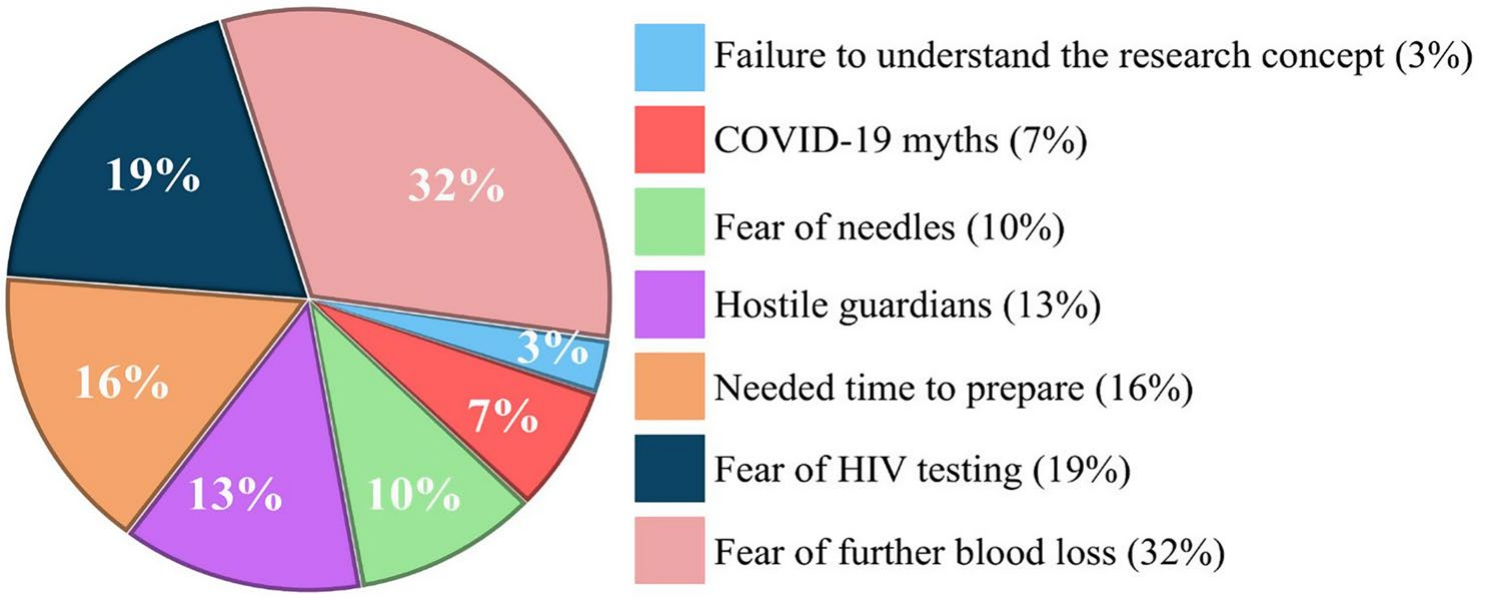
Participants’ reasons for refusing to take part in the study. Each slice of the pie represents a reason for non-participation, with the slice size proportional to the percentage of participants who reported that reason

We enrolled a total of 63 participants in the study. Over the four-week study period, the study team could only approach 94 of 213 eligible trauma patients due to enrolment occurring between 08:00 and 22:00 (Fig. 1). As 55.7% of patients arrived outside these hours, 119 eligible patients were missed. The overall uptake of combined HIV, HBV, and HCV testing among the study population was 67.02%. Thirty-one patients declined to participate in the study, most commonly due to fear of further blood loss, misconceptions related to COVID-19, and fear of needles (Fig. 2).

### Participant demographics and clinical characteristics

Among the 63 participants, 49 were males (77.8%). The average age of the study participants was 32 years. Most participants were manual workers (*n* = 26, 41.3%), married (*n* = 40, 63.5%) and had attained secondary school education (*n* = 30, 47.6%). A significant majority had no history of transfusion (*n* = 59, 93.65%) or HBV vaccination (*n* = 61, 96.8%), and none reported intravenous drug use. Most participants had one sexual partner (*n* = 47, 74.6%). The leading causes of traffic-related trauma were car (*n* = 12, 19%) and motorcycle accidents (*n* = 9, 14.3%), whereas non-traffic-related trauma cases were predominantly due to assault (*n* = 18, 28.6%). Most injuries presented were due to abrasions (*n* = 39, 61.9%). Emergency care was accessed within 2–4 h of injury by 60.3% (*n* = 38) of the participants (Table 1).

**Table 1:** Sociodemographic characteristics, risk behaviours and injury history among trauma psatients at QECH, 2021

Background Information	Crude *N*	%
**Age (years)**		
10–19	6	9.5
20–29	29	46.0
30–39	14	22.2
40–49	8	12.7
50–59	4	6.3
60 Above	2	3.2
Median age – 29		
IQR 17–65		
**Sex**		
Male	49	77.8
Female	14	22.2
**Occupation**		
Self Employed	13	20.6
Sedentary workers	6	9.5
Manual workers	26	41.3
Unemployed	18	28.6
**Marital status**		
Single	20	31.8
Married	40	63.5
Divorced	1	1.6
Separated	2	3.2
**Education**		
Primary	15	23.8
Secondary	30	47.6
Tertiary	18	28.6
**Religion**		
Christian	60	95.2
Muslim	3	4.8
**Transfusion history**		
Transfused	4	6.35
Not transfused	59	93.65
**HBV vaccination**		
Vaccinated	2	3.2
Not Vaccinated	61	96.8
**Intravenous drug use**		
YES	0	0
NO	63	100
**Number of sexual partners**		
None	3	4.8
1 Partner	47	74.6
2 or more partners	13	20.6
**Mechanism of injury**		
**Traffic related**	**23**	**36.5**
Bicycle accident	2	3.2
Car accident	12	19
Motorcycle accident	9	14.3
**Non-traffic related**	**40**	**63.5**
Assault	18	28.6
Collapsed Structure	7	11.1
Electrocution	1	1.6
Fall	8	12.7
Fallen body	6	9.5
**Type of injury**		
Abrasion	39	61.9
Laceration	18	28.6
Fracture	5	7.9
Stab wounds	1	1.6
**Clinical outcome**		
Admitted	4	6.35
Outpatient	59	93.65
**Time from injury to arrival at AETC (hours)**		
0–1	1	1.6
2–4	38	60.3
5–8	2	3.2
9–12	8	12.7
13–16	7	11.1
17–20	2	3.2
21–24	2	3.2
More than 24	3	4.8

### Targeted screening among trauma patients resulted in the detection of new HIV, HBV, and HCV cases

**Fig. 3:**
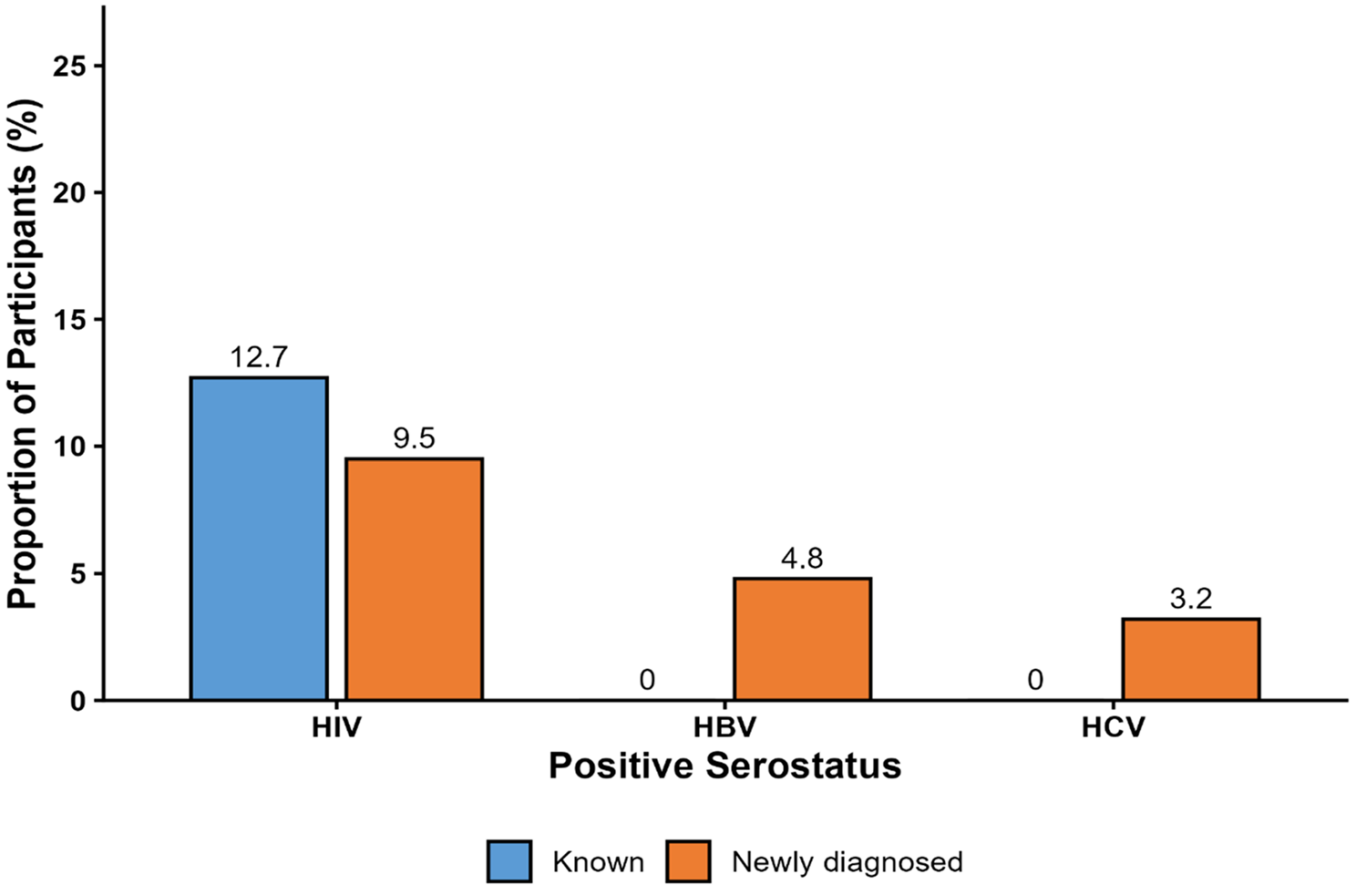
Case detection rates for HIV, HBV, and HCV among trauma patients. The bars represent proportions (%) of participants with known (blue) and newly diagnosed (orange) infections

**Fig. 4:**
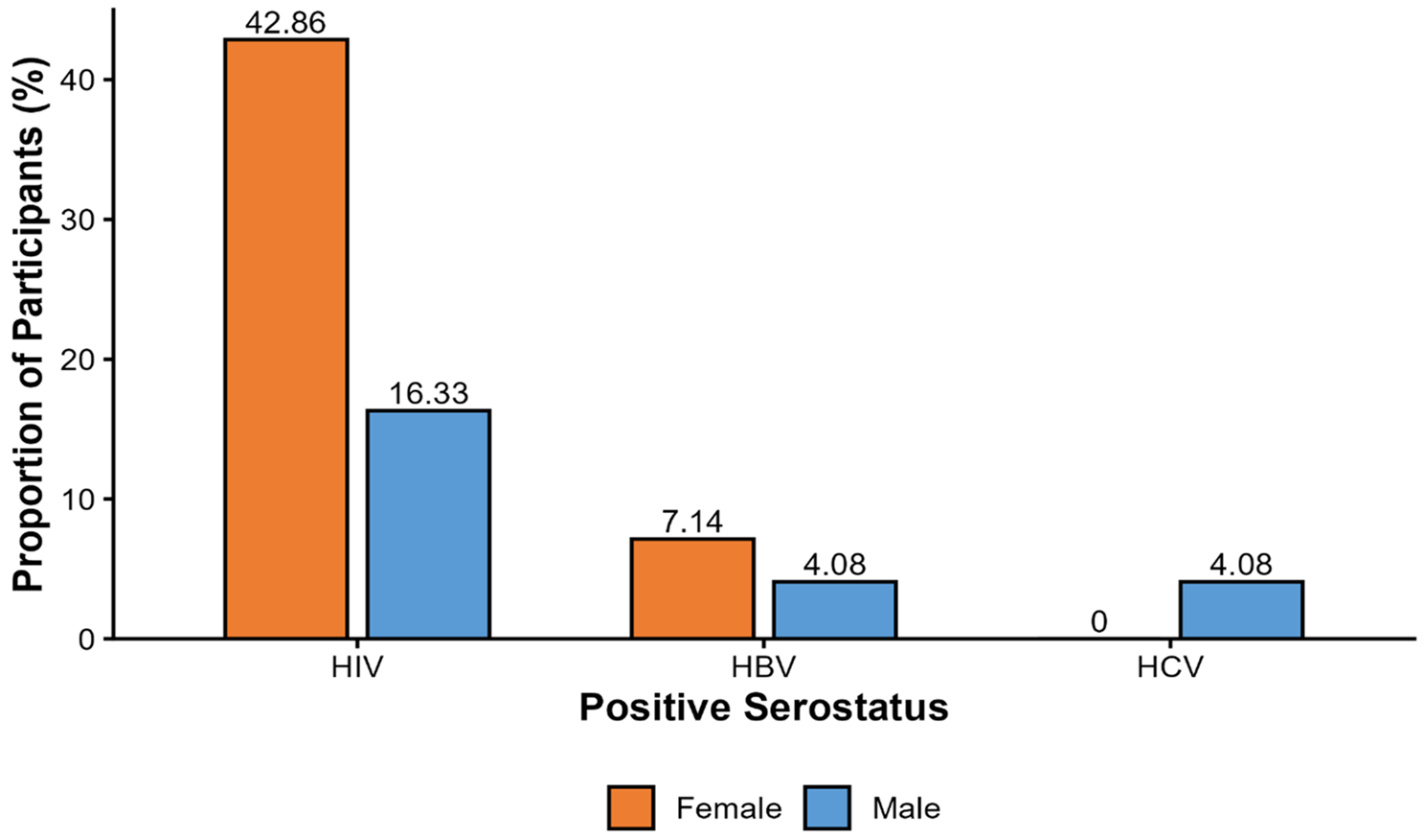
Case detection rate of HBV, HCV, and HIV by sex among trauma patients. Horizontal bars represent the percentage of patients testing positive for each infection within each sex

Of the 63 participants enrolled in the study, 6 were newly identified as HIV seropositive, while 8 individuals already knew their HIV-positive status before enrollment. Based on the proportion of newly diagnosed cases, the HIV case detection rate among participants was 9.5%, which was higher than that observed for HBV at 4.8% and HCV at 3.2% (Fig. 3). HIV positivity was more frequently observed among female participants (6/14, 42.9%) compared to males (8/49, 16.3%) (Fig. 4). Among females who tested positive for HIV, four had a prior diagnosis, while two were newly diagnosed. In contrast, half of the HIV-positive males (4/8) were newly diagnosed. Of the 3 participants who were seropositive with HBV, 2 were female, and one was male. Additionally, 2 participants tested positive for the hepatitis C virus (HCV), and both were male (Fig. 4). Only one individual (female) was found to be co-infected with both HIV and HBV. Importantly, all cases of HBV and HCV were newly diagnosed (Fig. 3), and there were no reported co-infections involving HBV and HCV or HIV and HCV.

### Seropositivity of HBV, HCV and HIV and their association with sociodemographic and clinical characteristics

HIV seropositivity was higher among females than males; however, this difference was not statistically significant (OR 3.8, 95% CI 1.0–14.1; Fisher’s exact *p* = 0.063). The wide confidence interval reflects imprecision, likely due to the small number of cases. No significant associations were identified between HIV seropositivity and age, nationality, marital status, occupation, educational level, intravenous drug use, or transfusion history. No statistically significant associations were observed between HBV seropositivity and any examined sociodemographic or clinical variables. HCV seropositivity occurred only among participants with primary school education (two cases); however, this finding was not statistically significant (Fisher’s exact *p* = 0.054). No other variables were significantly associated with HCV infection. Overall, none of the assessed factors demonstrated statistically significant associations with HBV, HCV, and HIV seropositivity, and findings should be interpreted cautiously given the limited number of positive cases.

## Discussion

This study evaluated the feasibility of HBV, HCV, and HIV testing among trauma patients who presented to the Adult Emergency and Trauma Centre at Queen Elizabeth Central Hospital. Rapid diagnostic tests were employed for initial screening due to their routine use in Malawian health facilities and operational suitability in emergency settings, with confirmatory enzyme-linked immunosorbent assay testing performed to ensure diagnostic accuracy.

In this cohort, 67.02% of injured patients approached by the study team consented to testing for HBV, HCV, and HIV, resulting in a refusal rate of 33%. This refusal rate is comparable to the 34% opt-out rate reported among Malawian outpatients offered facility-based HIV self-testing [18]. In that study, males were more likely to decline testing, and overall, the main reasons for refusal were feeling unprepared for testing (49.4%) and perceiving a low risk of HIV infection (30.4%). Similarly, our refusal rate likely reflects internalised barriers, including the need for more time to prepare for a potential positive result (16%), and fears associated with HIV testing (19%) or additional blood loss from venipuncture (32%). The predominance of male patients presenting to the trauma centre in our study also likely contributed to the refusal rate. This underscores the necessity of targeted and sustained community engagement campaigns to enhance uptake of blood-borne virus testing in our population.

The case detection rate for HIV observed in this trauma population was higher than that reported in comparable studies from the region, indicating a substantial occupational exposure risk for healthcare workers involved in trauma care [19–21]. Given the frequent contact with blood and bodily fluids in this setting, these findings underscore the importance of rigorous infection prevention and control practices and support the consideration of routine blood-borne virus testing in emergency departments. The proportion of new HBV and HCV cases was comparable to that reported in other trauma-based studies [22, 23], further supporting the rationale for systematic viral hepatitis testing approaches in this population. In comparison to previous reports from QECH, where stab wounds accounted for 7.3% of assault-related presentations, our study observed 1.6%, highlighting a shift in assault patterns [24].

HIV–HBV coinfection, although infrequent in this study, remains clinically important because of its association with accelerated liver disease progression and increased mortality [25–29]. The presence of coinfection reinforces the need for comprehensive blood-borne virus testing and highlights the importance of hepatitis B vaccination among healthcare workers as a key preventive strategy aligned with global elimination targets.

Importantly, a substantial proportion of infections identified in this study were previously undiagnosed, indicating that provider-initiated testing in the emergency trauma setting could be effective at detecting unrecognised infections. This also suggests that injured patients represent a unique population who may not otherwise seek care, as most are generally healthy before the injury. Implementing routine testing in this setting would therefore offer an important opportunity to detect unsuspected viral infections and enable timely linkage to care. Evidence from comparable settings indicates that individuals diagnosed early are more likely to achieve viral suppression and favourable immunological outcomes, highlighting the public health and clinical benefits of early case detection and linkage to care [30–32].

In this study, no statistically significant associations were observed between HBV, HCV or HIV seropositivity and the assessed sociodemographic or clinical variables. HIV seropositivity was more frequent among females compared to males, consistent with established biological and sociobehavioral vulnerabilities and national estimates from the Malawi Population-based HIV Impact Assessment, which reported higher HIV prevalence among women (10.5%) than men (7.1%) [33]. Conversely, HBV and HCV seropositivity were more frequently observed in males, in line with findings from studies of Malawian blood donors that reported higher rates of these infections among men [34]. HCV seropositivity in our cohort occurred only among participants with primary-level education. However, these observations were based on small numbers of positive cases and imprecise estimates. The limited statistical power restricts definitive inference, and larger studies are warranted to clarify potential subgroup differences.

We acknowledge several limitations in our study. The relatively small sample size restricted the precision of estimates and limited robust inferential analyses; therefore, our analysis was limited to descriptive statistics. Enrolment was confined to operational hours (08:00–22:00, excluding weekends), during which we approached 94 out of 213 eligible trauma patients. As a result, 119 eligible patients (55.7%) who presented outside these hours were not enrolled, which may have introduced selection bias. Additionally, the occasional presence of guardians during recruitment may have influenced patients’ decisions to refuse to enrol into the study. Patients with reduced consciousness or more severe injuries were also excluded from enrolment, which may have further contributed to selection bias. These factors should be considered when interpreting the findings.

## Conclusion

This study reports a 67% uptake among trauma patients who were offered provider-initiated testing and identifies previously undiagnosed cases of HBV, HCV, HIV, and HIV–HBV coinfection. These findings demonstrate that such testing can be effectively implemented in an emergency trauma setting and is successful in detecting new infections. Although uptake was limited by the opt-in nature of the testing strategy and limited enrolment hours, the results highlight the potential value of strengthening structured provider-initiated testing strategies within trauma care settings. Adopting more routine or opt-out testing strategies may further enhance early diagnosis, timely linkage to care, reduce transmission, and improve occupational safety for healthcare workers in high-burden settings.

## Supplementary Information

Below is the link to the electronic supplementary material.

Supplementary Material 1

## Data Availability

All the data generated or analysed during this study are included in this published article.

## Abbreviations

HBV: Hepatitis B Virus
HCV: Hepatitis C Virus
HIV: Human Immunodeficiency Virus
AETC: Adult Emergency and Trauma Centre
QECH: Queen Elizabeth Central Hospital
ELISA: Enzyme-Linked Immunosorbent Assay
