# Supplementary material for: Acceptability of HBV, HCV, and HIV screening among injured patients presenting to the adult emergency department in Blantyre, Malawi
Source: BMC Infect Dis. 2026 Mar 19;26:842. doi: 10.1186/s12879-026-13120-0 (PMC13123122; doi:10.1186/s12879-026-13120-0)
Supplement: Supplementary file 1 — Supplementary Material 1 [file 12879_2026_13120_MOESM1_ESM.pdf]

INJURY QUESTIONNAIRE

Instructions:

For most questions, there is a choice of answers. Simply pick the one that's true for you by Ticking in the box. There are some questions where you need to write in an answer. For these questions, a space will be provided for you. It is important that you answer every question as best as you can. There are no right or wrong answers, we just ask you to be completely honest.

a. PERSONAL DETAILS

a. Participant ID...

b. Age..

c. Sex

M ☐ F ☐

d. Nationality

Malawian ☐ International ☐ Specify...

e. Occupation..

f. Marital status

Single☐ Married ☐ Divorced☐ Separated☐ Widow/widower ☐

g. Level of Education

None ☐ Primary ☐ Secondary ☐ Tertiary☐

b. TRANSFUSION HISTORY

a. Have you ever been transfused with blood?

Yes☐ No☐

c. VACCINATION HISTORY

a. Have you been vaccinated for Hepatitis B?

Yes☐ No☐

d. DRUG USE

a. Have you ever done intravenous drug use?

Yes☐ No☐

e. SEXUAL PARTNERS

a. How many sexual partners have you had in the past 12 months?

1☐ 2☐ 3☐ 4☐ >4 ☐

f. MEDICAL HISTORY

a. Have you been previously tested for:

Hepatitis B☐ Hepatitis C ☐ HIV☐

b. Have you tested positive for any of the above?

No☐ Yes☐ Specify…………………………………………………

g. TRAUMA HISTORY

a. Date of Injury: ……………………………………………………………….....

b. Time of Injury: ……………………………………………………… (24hr time)

c. Where did injury occur? Address/district (be specific) …………………………………...

Location type: Home☐ Work ☐Road / Street ☐School ☐Farm ☐

Sports/Recreation ☐ Public Building☐

Other…………………………………………

d. Mechanism of Injury:

- **Traffic Related**: Pedestrian ☐ Bicyclist ☐ Motorcyclist☐ Driver ☐ Passenger ☐ Passenger on Bicycle ☐ Passenger on motorcycle ☐
- **Non-Traffic Related**: Animal Bite☐ Human Bite ☐ Gun Shot Wound ☐ Foreign Body☐ Fall _______ meters Burn by ____________, % Body area _________ Assault with _________ Collapse☐tructure Other _______________________

e. Was the patient intentionally injured by another?

No☐ Yes☐ Unknown☐

f. Injury sustained: Type ………………. Location………………… (give all injuries identified in patient.

	TYPE		LOCATION		LOCATION
0	Contusion	0	Head or Skull	14	Hip
1	Laceration	1	Face, Ears, Eyes, Nose	15	Thigh or Femur
2	Abrasion	2	Neck or Cervical Spine	16	Knee
3	Fracture	3	Shoulder or Clavicle	17	Leg or Tib/Fib
4	Bite	4	Arm or Humerus	18	Ankle
5	Burn	5	Forearm or Radius / Ulna	19	Foot
6	Penetrating Wound / Stab	6	Wrist	20	Toes
7	Dislocation	7	Hand	21	Elbow
8	Gun Shot Wound	8	Fingers	22	Other_______________
9	Injury to Internal Organ	9	Chest, Thoracic Spine, Ribs		
10	Head Injury	10	Abdomen or Lumbar Spine		
11	Spine Injury	11	Flank		
12	Foreign Body	12	Pelvis		
13	Other______________	13	Buttocks		

g. Clinician Diagnosis: ……………………………………………………..

h. Outcome: Outpatient care ☐ Admitted ☐
